# The memory we miss: exploratory behavior as a window into developmental recognition processes

**DOI:** 10.3389/fnbeh.2026.1919577

**Published:** 2026-10-13

**Authors:** Eleanor G. Williamson, Jesse D. Cushman, Patrese A. Robinson

**Affiliations:** 1 Neuroscience Program, Bryn Mawr College, Bryn Mawr, PA, United States; 2 Neuroscience Program, Psychology Department, Haverford College, Haverford, PA, United States; 3 Neurobehavioral Core, Neurobiology Laboratory, National Institute of Environmental Health Science, Durham, NC, United States

**Keywords:** affordances, behavior, developmental learning, exploration, rodent, recognition memory, spatial memory

## Abstract

Recognition memory tasks are among the most widely used approaches for studying learning and memory across the lifespan, yet the behavioral measures used to assess memory, particularly during developmental studies, remain limited. In most rodent studies, recognition memory is inferred almost exclusively from discrimination ratios or indices that quantify preferential exploration of novel stimuli. While these measures are valuable, their widespread use may constrain our understanding of the cognitive-behavioral processes supporting nuanced memory expression across ontogeny. Similar concerns have emerged in fear conditioning, where expanding analyses beyond freezing behavior revealed previously unrecognized differences in memory. In this perspective, we argue that recognition memory research would benefit from expansion of behavioral analyses. Drawing on affordance-based approaches to object exploration, we discuss evidence showing that object properties influence memory performance and exploratory behavior. Importantly, our recent findings indicate that pre-weanling (but not adult) rats exhibit task-dependent shifts in exploratory behavior, contingent on the object’s affordances, which are not captured by traditional discrimination measures. These findings suggest that developmental differences in memory may be expressed not only through recognition performance but also through the exploratory strategies animals employ during retrieval. Expanding behavioral analyses in recognition tasks may therefore reveal developmental neurobehavioral mechanisms that remain hidden from conventional metrics, providing a more complete understanding of memory development. The challenge moving forward is not simply to determine whether young animals remember, but to identify how they remember and whether the behavioral routes to successful memory change across development.

## Introduction

Novel object recognition (NOR) and location (NOL) tasks capitalize on rodents’ innate novelty preference and have revealed significant corticolimbic contributions to memory that are dependent on both the psychological (e.g., identity versus spatial) and temporal (e.g., short-term versus long-term memory) demands of the task (Antunes and Biala, 2012; Barker et al., 2007; Barker and Warburton, 2011; Bernstein et al., 2019; Cruz-Sanchez et al., 2021; Dix and Aggleton, 1999; Ennaceur and Delacour, 1988; Jablonski et al., 2013; Mumby et al., 2002; Reger et al., 2009a; Stevanovic et al., 2022; Warburton and Brown, 2015). Further, NOR and NOL are well-established paradigms for studying the behavioral ontogeny of memory (Ainge and Langston, 2012; Asiminas et al., 2022; Bath et al., 2017; Contreras et al., 2019; Cruz-Sanchez et al., 2020, 2021; Jablonski et al., 2013; Langston and Wood, 2010; Ramsaran et al., 2016a, 2016b; Reger et al., 2009a; Silvers et al., 2007; Westbrook et al., 2014). The intrinsic novelty preference that underlies object recognition assays likely has overlap with reinforcement mechanisms engaged when external rewards are presented by the experimenter. For example, access to novel objects has the capacity to condition a place preference, suggesting novelty serves as its own internally generated reinforcement signal (Bevins et al., 2002). However, these paradigms offer a procedural advantage over classical or operant conditioning paradigms; they do not rely on external reinforcers (e.g., shocks or food), which may influence regional circuit recruitment and obscure *age-dependent* learning-related neurobiological changes (Antunes and Biala, 2012; Ennaceur and Delacour, 1988; Lueptow, 2017; Reger et al., 2009a; Silvers et al., 2007). A rich repository of information about the psychobiological processes underlying object identity and spatial memory (using NOR and NOL, respectively) exists, and using these simple forms of recognition tasks to assess cognitive function at various life stages or following neurobiomolecular manipulations (e.g., developmental stress, genetic models of disease, drug exposure/use) has revealed their sensitivity to and utility for translational applications (Bath et al., 2017; Brown et al., 2016; Calamandrei et al., 2002; Khandelwal et al., 2023; Reichel et al., 2014; Reincke and Hanganu-Opatz, 2017; Schwabe et al., 2024; Stangherlin et al., 2009).

While useful, these tasks and their outcomes are often oversimplified, potentially overlooking more nuanced cognitive processes. Researchers should consider practices that standardize interpretation (e.g., use of capacitive sensor objects to better quantify exploration; Spry et al., 2021), recognize which measures are prone to misrepresentation, and be open to extensive behavioral analyses that demonstrate dissociable neurobehavioral outcomes per task. This last point is especially true for *assessing memory ontogeny*; our previous work using fear conditioning demonstrated that young animals have distinct memory-related behavioral patterns from adults (Jablonski et al., 2012; Robinson and Stanton, 2015; Sanders et al., 2020; Stanton et al., 2021) and different neurobiological (Asok et al., 2013; Robinson et al., 2018) contributions to similar behaviors in adults that, if not taken into consideration, would lead to misinterpretation of memory (or the lack thereof). In contrast, even a cursory review of recognition memory studies reveals limited behavioral measures for characterizing how memory-related behaviors change as animals develop (see Table 1 for representative publications). Here, we argue that recognition tasks across development should routinely include and report a broader range of metrics. Expanding behavioral assessment can better capture neurobehavioral distinctions throughout ontogeny and provide richer interpretations of behavior than traditional recognition memory measures alone.

**Table 1:** A brief reference list of common recognition task procedures.

Citation	Task	Species	Strain	Testing Age(s)	Handling	Habituation	Primary Discrimination Measure	Other Exploratory Behavior Measures	Definition of Object Interaction/Exploration	Object Type + Specificiations
Ainge and Langston (2012)	NOR, NOL	Rat	Lister Hooded	PD24,30	2 min/day, 3 days.	15 min (group) + 5 min (individual), day prior	(Tnovel-Tfamiliar)/Ttotal	Ttotal (sample)	Nose directed at object and sniffing or whisking within 2 cm of object	Various objects: non-porous, flat base, larger than animals in one dimension
Albasser et al. (2010)	*NOL	Rat	Dark Agout (Males Only)	12–14 weeks adults	Not stated	7-day Bowtie Maze food well baiting	(Tnovel-Tfamiliar)/Ttotal (test phase)	Ttotal (all phases)	Directing the nose at a distance < 1 cm to the object and/or touching it with the nose or the paws	Novel = 126 distinct objects, Familiar = 21 distinct objects, Object type not specified
Bardgett et al. (2011)	NOR	Mouse	APPTg2576 (B6SJLF1/J x C57B16/J background)	12–14 months	None stated	15 min/day, 3 days	Tnovel-Tfamiliar	Ttotal (test)	Within 2 cm of object and oriented within 45 degrees of object; paw on object	Six “groups” of objects (glass or plastic)
Barker and Warburton (2011)	NOR, NOL, OiP	Rat	Male Dark Agouti	PD17,21,26,31	Once daily, 1 week	10-15 min/day, 4 days	(Tnovel-Tfamiliar)/Ttotal	Ttotal (sample + test)	Nose directed at object within 2 cm	Duplo (Lego) blocks: various shapes, colors, sizes (9 × 8 × 7 to 25 × 15 × 10cm)
Bekci et al. (2024)	NOR, NOL	Rat	Sprague–Dawley, Nictotine-preferring SD	3–4 months	None stated	5 min, day prior to NOR (NOL done 1 week after NOR)	(Tnovel-Tfamiliar)/Ttotal	Ttotal (sample), Tfamiliar vs. Tnovel (test)	Sniffing object or tilting head less than 2 cm from object	“Objects were different enough for animals to distinguish between but not so different as to make them avoid one object”
Broadbent et al. (2010)	NOR	Rat	Long-Evans (Males only)	300-350 g at beginning of study	None stated	5 min/day, 2 days	Tnovel/Ttotal (%; first 15 and 30s of test, all of test)		Nose within 1 cm of object and vibrissae moving	Ceramic or plastic objects, various colors and sizes (7.6–8.9×7.5–12.7 cm)
Calamandrei et al. (2002)	*NOR + OiP	Mouse	CD1 Mice	PD28,45,70	None stated	4 min, day of	Difference in each object’s exploration time between “sample” and “test” sessions	Tnovel vs. Tfamiliar between “sample” and “test” sessions; Ttotal in “habituation”; crossing arena, grooming, rearing + top rearing (all sessions)	“In contact” with object	Parallelepiped (3 × 3 × 4cm); L-shaped wall (7 × 7 × 4cm); cube (5 × 5 × 3cm); H-shaped platform (9 × 8 × 2.5 cm), Novel Object: Stair (7 × 6 × 4.5 cm)
Cruz-Sanchez et al. (2020)	NOR, NOL	Mouse	C57/129 J	PD16,21,25,28,35	5 min × 2/day, 4 days	4 min x2/day, 4 days	Tnovel/Ttotal (first 20s of exploration)	Tfamiliar vs. Tnovel	Object-directed gaze with sniffing/pawing within 2 cm of object	3D printed step (46x46x48mm) or dome (47 × 48mm)
Guilhaume‐Correa et al. (2020)	NOR	Mouse	C57BL/6 J (Males Only)	~PD46,58,60,72	None stated	none stated	Tnovel/Ttotal	Distance traveled, mean velocity of movement, center time + frequency	Touching, climbing, smelling or facing object closely	Not stated–cite Bevins and Besheer (2006) which is a general NOR protocol
Herring et al. (2008)	NOR, NOL	Rat	Sprague–Dawley (Males Only)	~PD56	None stated	10 min/day, 3 days. Stereotypy + locomotion measured before habituation; NOR then NOL next day	Tnovel/Ttotal		Nose within 1 cm of object and vibrissae moving	20 object types: all “sized appropriately”
Krüger et al. (2012)	NOR, *NOL, temporal order	Rat	Wistar (Male Only)	P15-17	None stated	10 min (included objects not used in NOR)	Tnovel/Ttotal	Grooming, rearing, wall-rearing, defecation/urination (habituation)	Entry of head into 2 cm diameter of object, not including climbing or sitting on object	Six pairs of “differently shaped and textured, easy to clean items” that were climbable
Kwapis et al. (2020)	*NOL	Mouse	C57BL/6 J (Males Only)	2–4 months or 18–20 months	2 min/day, 4 days	5 min/day, 6 days	(Tnovel-Tfamiliar)/Ttotal (all phases)	Ttotal (all phases)	Head/nose oriented toward object and within 1 cm of it	200 mL glass beakers filled with cement
Mumby et al. (2002)	NOR, NOL, OiC	Rat	Long-Evans	PD70-84	None stated	20 min/day, 3 days 1 week prior (included objects not used in actual test)	Tnovel/Ttotal	Ttotal (sample, test)	Head oriented within 45 degrees and 4 cm of object; rearing with forepaw on object	18 different objects: metal, glass, porcelain, or glazed ceramic (4.5–15 × 4-10 cm)
Pattwell et al. (2011)	*NOL	Mouse	C57BL/6 J (Males Only)	PD29,39,49	None stated	5 min, day prior	Tnovel/Ttotal	Avoidance of center + preference for one region of arena, locomotion + “exploratory behavior” (sample)	None stated; refer to three different labs for general task description	Lego block trees (4.8 × 4.8x7cm)
Reichel et al. (2014)	OiP	Rat	Long-Evans (Males Only)	250-300 g on day of delivery	Daily, 4 days	5 min/day, 2 days	Tnovel/Ttotal		Sniffing or touching object with nose, not when sitting, leaning, or standing on object	PVC pipe (6.4 × 3.8 cm^2), paint roller (7.5 × 2.5 cm^2), light bulb (8.9 cm), or plastic bottle (12 cm)
Saenno et al. (2022)	NOR, NOL	Rat	Sprague–Dawley	~PD56	None stated	30 min, day prior	Tnovel/Ttotal (% time with novel)	Tnovel vs. Tfamiliar (sample + test); distance moved (habituation, sample)	Chewing, licking, sniffing, or pointing nose towards objects within 2 cm	Not stated
Schwabe et al. (2024)	NOR, NOL	Mouse	E3FAD or E3/4AD (Females Only)	6–7.5 months	None stated	5 min/day, 2 days; 2 min immediately prior to sample	Tnovel (compared to chance value of 15 s (1/2 total time))	Time taken to reach 30s of exploration	Nose in contact with object	Not stated; prior work in lab used LEGO Duplaco block(s)
Siegel et al. (2011)	NOR, NOL	Mouse	C57BL/6 J	~PD32 (tested on OFT + EZM prior)	None stated, handled for previous testing	5 min/day, 3 days	Tnovel/Ttotal (% time with novel)	Ttotal (across trials), Tnovel-Tfamiliar	Nose within object zone	Plastic toys: man, monkey, horse cow
Stangherlin et al. (2009)	NOR	Rat	Wistar (Male Only)	PD28	None stated	5 min, day prior	Tnovel/Ttotal (% time with novel)	Tnovel/Ttotal (“training”)	Sniffing or touching object with nose and/or forepaws	Triangle, ball, or rectangle (10 × 10cm), various colors
Sun et al. (2010)	NOR, NOL	Rat	Fischer	>PD60	None stated	5 min (without objects); NOL with retention of 1,10,60, or 120 min and NOR with retention of 10 or 60 min before reported tests	Tnovel/Ttotal	Tnovel vs. Tfamiliar	Sniffing, whisking, or looking at object within 2 cm	NOR: various bottles or cans, NOL: “more intricate. and complex”
Westbrook et al. (2014)	NOR, NOL	Rat	Long-Evans	PD17,21,26	3 min before each habituation session	10 min/session, 3 sessions (day before + of test)	Tnovel/Ttotal	Ttotal (sample)	Actively sniffing, pawing at, or whisking with snout directed towards object	Fake green apple (7 × 7cm), glass jar with stones inside (8.9 × 7.6 cm), white hook (7.6 × 8.9 cm)

Summary of common methodological variations in Novel Object Recognition (NOR), Novel Object Location (NOL), and related behavioral paradigms across select published literature. Beyond the fairly standard *habituation phase → sample encoding phase → retrieval test phase* paradigm structure, researchers alter nearly every experimental variable, including handling durations, habituation times, and specific behavioral definitions. There is no universal consensus on what constitutes object interaction, and behavioral inclusion criteria span a wide spectrum, including *Proximity-based inclusion metrics* (i.e., strict distance boundaries ranging from “in contact” to within 1 cm, 2 cm, or 45 degrees of the snout) and *Anatomical markers-based inclusion metrics* (e.g., specify nose-directed interaction, sniffing, whisking, or pawing). The physical properties (e.g., materials or geometric complexity) of the objects used vary widely, which can strongly influence an animal’s natural preferences or emotional responses to them. The primary mathematical measures used to assess memory or discrimination performance can be inconsistent and typically unimaginative in how they consider the many ways exploration data *could* be assessed. To establish reliable developmental benchmarks, researchers must implement standardized, age-adjusted protocols that utilize consistent mathematical indices and cross-species definitions of exploration. Furthermore, future studies should employ universally categorized stimulus objects alongside uniform habituation windows to ensure that performance metrics reflect true memory capacity rather than differences in motor control or environmental stress.

## Behavior: A window into developing neurocircuitry

A brief overview of recognition neurocircuitry in adult rodents reveals a primary circuit of task-specific corticolimbic and temporal networking. In adult rats, perirhinal cortex (PRH) supports object identity memory across a range of retention intervals (Barker and Warburton, 2011; Mumby et al., 2007; Warburton and Brown, 2015; Winters and Bussey, 2005). However, it is generally agreed (Cohen and Stackman, 2015) that, while the hippocampus (HPC) is responsive to novelty (Brown et al., 2020), it is only critical for object identity memory following long (>1 h) retention intervals (Ainge et al., 2006; Chao et al., 2022; Clark et al., 2000; Cohen et al., 2013; Hammond et al., 2004). In contrast, spatial recognition memory critically depends on a functional HPC (but not PRH) across all retention intervals (Barker and Warburton, 2011, 2015; Mumby et al., 2002). Variations of the recognition tasks that assess associative spatial-identity memory, contextual memory, and temporal-order recognition demonstrate a critical role for and connections between the medial prefrontal and retrosplenial cortices with the perirhinal and hippocampal cortices as psychological demands (i.e., the type of associative information acquired and retrieved) of the task change (Barker and Warburton, 2011, 2015, 2020a, 2020b; de Landeta et al., 2020, 2021; Haijima and Ichitani, 2012; Mumby et al., 2002; Norman and Eacott, 2005; Warburton and Brown, 2015). Regional activation studies extend this network and further specify task-specific contributions of the HPC subfields (i.e., dentate gyrus), thalamus, hypothalamus, temporal lobe area TE, and the entorhinal cortex (Albasser et al., 2010; Arias et al., 2015; Barbosa et al., 2013; Bernstein et al., 2019; Mendez et al., 2015; Polanczyk et al., 2026; Weible et al., 2009; Zhu et al., 1995, 1996). Together, these results in *adults* reveal a psychologically dissociable set of tasks with distinct neuroanatomical circuitry, shaping our understanding of the behavioral neurobiology of recognition learning.

While less is known about the roles of these brain structures during ontogeny, behavioral measures help infer their neurobiological involvement in object and spatial recognition across development. Using *discrimination ratios* (DR: novel object exploration relative to total object exploration) or *discrimination indexes* (DI: novel–familiar exploration difference relative to total exploration) as memory measures, short-term NOR memory (<1 h) emerges around postnatal day (PD)14, whereas NOL memory becomes stable during the third postnatal week (>PD19; for review, see Cruz-Sanchez et al., 2021). Long-term (24 h) memory emerges during the fourth postnatal week in both tasks. Together, these findings suggest that PRH-supported identity discrimination emerges after the second postnatal week, whereas HPC-dependent spatial recognition and long-term retention mature during the third and fourth postnatal weeks, respectively. While compelling, these developmental profiles only infer neuroanatomical recruitment from adult findings and rely largely on DR/DI measures to verify memory.

Conventional behavioral measures may obscure developmental differences in neurocircuit recruitment. An exemplary shift has emerged in fear-conditioning studies, in which freezing has traditionally been the primary measure of fear memory. Fear conditioning paradigms are valuable comparison points for novelty recognition tasks because they have well-characterized developmental dissociations in behavior (Stanton, 2000; Stanton et al., 2021), engage overlapping neurocircuitry with recognition tasks (Callaghan et al., 2019; Hunt et al., 2007; Stanton et al., 2021), and have a considerably longer research history, providing a century-long body of work from which to draw inferences about the developmental processes underlying recognition memory that was first formally characterized in rodents in the 1980’s (Ennaceur and De Souza Silva, 2018; Ennaceur and Delacour, 1988).

Recently, darting, an active escape-like response, has been identified as a psychologically and neurobiologically meaningful learning-related behavior (Gruene et al., 2015; Nieves et al., 2023; Trott et al., 2022). Mitchell et al. (2024) showed that although male and female rats freeze reliably during tone-fear conditioning, conditioned darting occurs more frequently in females. Importantly, darting was associated with differences in lateral/basolateral (l/bl) Amygdala and lateral (l)PAG c-Fos expression during conditioning. Overtraining shifted males toward darting behavior (Mitchell et al., 2022), suggesting delayed recruitment of PAG/AMG circuitry relative to females. Here, combining freezing and darting captures neuroanatomical recruitment better than freezing alone. Similar combinatorial approaches could reveal juvenile and adolescent PAG/AMG activity that is distinct from that of adults (Perry et al., 2025; Zimmermann et al., 2023) and indicates unique neurobehavioral learning processes. Considering this example, an extended analysis of previously disregarded behaviors in recognition tasks could further enrich our understanding of developmental memory processes.

## Recognition memory needs more than novelty preference

In their review, Blaser and Heyser (2015) emphasize the utility of recognition memory tasks as a tool for translational research in non-human animals while highlighting inadequacies in procedural standardization across models, particularly in accounting for species differences in behavioral responses to novelty or contextual modulation of exploratory behavior. These procedural and, specifically, analytical inadequacies also extend to developmental comparisons. Between weaning and adulthood, environmental exploration increases (Lynn and Brown, 2009), and rodent behavioral response to environmental/contextual stressors can change nonlinearly (Lin and Wilbrecht, 2022; Lynn and Brown, 2009; Macrì et al., 2002; Pattwell et al., 2011). Unfortunately, reports still rarely developmentally justify their choice of parameters and analyses. At best, objects or environments (e.g., arena dimensions) are scaled down at younger ages despite work indicating these factors’ influence on exploratory behavior (Baron et al., 2025; Inayat et al., 2021; Reger et al., 2009a) and calls to scale object size to animal size (Ricceri et al., 2000; Travaglia et al., 2018). Overall, measures of exploratory behavior typically do not account for potential developmental differences in behavior.

While procedural variability exists across species, *rodent* recognition memory paradigms use relatively standardized protocols (habituation → sample encoding → retrieval test) and primary measures of learning (i.e., raw exploration time, DR/DIs; Table 1). However, additional behavioral measures that may offer insight into *how* subjects learn are not consistently reported. Some reports include overall locomotion, rearing, climbing, grooming, and behavioral batteries (e.g., Calamandrei et al., 2002; Krüger et al., 2012; Shan et al., 2023, 2025) but this is far from standard practice. Further, even though behavior change during habituation sessions is a strong indicator of later NOR performance (Brown et al., 2016), investigating habituation phase behaviors is even more rare. Including a broader, consistent set of behavioral measures across all phases, alongside raw performance, would not only clarify discrepant results across laboratories but may also supplement explanations of why subject groups differ in exploration and object preferences.

The Arruda-Carvalho group comprehensively reviewed the ontogenetic emergence of recognition memory processes (Cruz-Sanchez et al., 2021). Their overview supports a framework in which developmental profiles of recognition memory reflect the maturation of what, what–where, and what-where-when memory systems. This progressive emergence was best characterized by the Stanton lab using a range of recognition paradigms (Buban, 2017; Jablonski et al., 2013; Ramsaran et al., 2016a, 2016b; Sanders et al., 2020; Westbrook, 2014; Westbrook et al., 2014). These studies demonstrate the protracted development of spatial recognition, associative, temporal-order effects, and the shift from short- to long-term retention. While particularly insightful in their descriptions of psychological phenomena, most report only exploration duration and/or frequency and DR/DIs as memory measures. Including multiple behavioral measures in recognition memory analyses could provide an extensive behavioral scaffold for the psychobiological frameworks of memory reported in these studies.

## Why objects matter: Affordances and recognition behavior

Training contexts (and the objects in them) shape responses and can reveal otherwise unnoticed behavioral differences. Studies highlight the role of visual, including object-specific, features in contributing to rodents’ spatial navigation and memory in recognition tasks (Arain and Cohen, 2013; Barker and Warburton, 2020b; Connor and Knierim, 2017; Deshmukh and Knierim, 2011; Keshen and Cohen, 2016; Komorowski et al., 2009; Muessig et al., 2015; Roullet et al., 1998; Schnell et al., 2023; Vollmer-Conna and Lemon, 1998; Wood et al., 2000) and how rodents integrate non-spatial with spatial features to encode a representation of a space (Connor and Knierim, 2017; Hayes et al., 2007; Keshen and Cohen, 2016). In cued fear conditioning, training stimuli are discrete, measurable features that are easily standardized and typically produce stereotyped, reproducible behavioral responses; recognition tasks, by contrast, often use a variety of (environmental) stimuli that are poorly standardized and can elicit a range of behavioral responses from subjects. Debate over how environmental features shape animal behavior has a long history (Chemero, 2003; Saborido and Heras-Escribano, 2023). Consideration of affordances (i.e., the relations between animal abilities and environment features; Chemero, 2003; Chemero and Heyser, 2005) has informed ecological, sociocultural, and biological accounts of perception and action in humans and non-human animals (Chemero, 2003; Chemero and Heyser, 2005; Rietveld and Kiverstein, 2014; Saborido and Heras-Escribano, 2023). Importantly, an animal’s *behavior is a direct consequence of its environmental affordances*. For object recognition tasks, the objects themselves dictate the exploration or memory behaviors displayed (Inayat et al., 2021). Therefore, fully characterizing these behaviors requires a careful examination of the specific object-related behavioral responses.

It is well-established that objects’ shape, size, and material influence rodents’ interactions with them (Chemero and Heyser, 2005; Heyser and Chemero, 2012; Inayat et al., 2021; Reger et al., 2009a; Tropea et al., 2022). Two decades ago, Chemero and Heyser (2005; Heyser and Chemero, 2012) urged neuroscientists to control for object affordances, warning that ignoring object affordances leads to poor neuroscience, critiquing “ruthless reductionism” (Chemero and Heyser, 2005, p. 419) that overlooks ecological significance in favor of neuromolecular study. They demonstrated that, during short-term NOR, mice spent more time exploring flat-topped climbable objects, habituated more slowly, and showed stronger recognition memory compared to mice exposed to touch-only objects. Despite criticizing researchers for failing to center methodologies around object ecological significance, Chemero and Heyser ironically omitted images or descriptions of their own touch-only objects. Anyway, the broader literature has still not heeded their call for rigorous affordance assessment. However, object variability across labs and within experiments may still create an opportunity for deeper neurobehavioral analyses of learning.

## What *memory* is missed: A developmental case study of object affordance

Measuring recognition memory primarily through DR/DI may mask learning because objects constrain behavioral expression, causing potential misinterpretations in developmental psychobiology. Recently, our team began manipulating object features during NOL testing to evaluate how environmental affordances shape developmental spatial memory ontogeny (Williamson et al., 2026). Using the Stanton lab protocols (Jablonski et al., 2013; Westbrook et al., 2014; Figures 1A,B), we examined ontogenetic differences in short-term (5 min retention) spatial recognition memory using 3D-printed objects (scaled to adult or pup size) that were asymmetrical and climbable/sittable (Flat/Asym; Figure 1C, left) or non-climbable/non-sittable and radially symmetrical (Pointed/Sym; Figure 1C, right). The Flat/Asym object provides spatially relevant orientation cues by having a specific left–right, front-back structure and a flat, climbable top surface. In contrast, the Pointed/Sym object impeded climbing and had no clear spatially orientable features. In this within-subjects (for age) design, our initial analysis of DRs (Figure 1D) revealed that *rats tested as pre-weanlings (~PD21) and adults* showed similar short-term spatial memory for the Flat/Asym but no memory using the Pointed/Sym, indicating that spatial learning was similarly facilitated at both ages when climbing was afforded. Most reports would stop there and suggest no age-related differences in memory.

**Figure 1:**
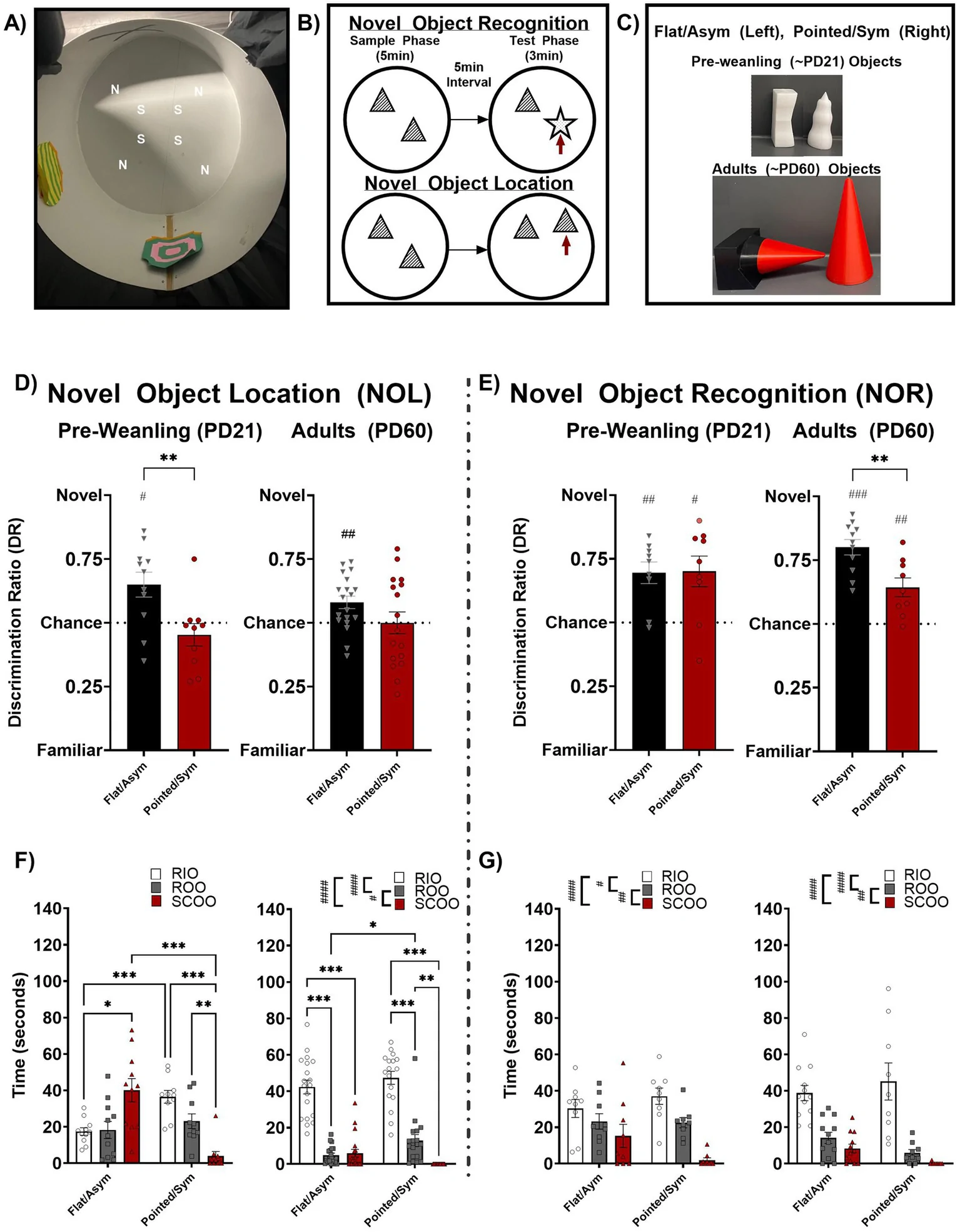
Diagrams of Arenas, Task Designs, and Objects Used in Behavioral Testing. **(A)** Top-down view of circular white polyester, wood, and resin arena used for tasks at both ages. For both NOR and NOL, sample phase locations are marked with “S.” During NOL test, novel test locations are marked with “N.”**(B)** Diagram of task procedures. During the 5-min sample phase, two identical objects were secured to the arena floor with magnets. After a 5-min retention phase, a 3-min test followed with either one familiar object exchanged for a novel object in its location (NOR) or one familiar object moved to a novel location (NOL). **(C)** A within-subjects design was used whereby all animals were tested at PD21 in either the NOL or NOR task and retested as adults in the other task not experienced at PD21. Male and Female Long-Evans rats were divided into training with either the (left) Flat/Asym or (right) Pointed/Sym object at about (top) ~ PD21 or (bottom) young adulthood young adulthood (~PD60). Sex × Orientation ANOVA, with discrimination ratio as the dependent variable, did not reveal any significant main effect (*p*’s > 0.44) or interaction effects (all *p*’s > 0.24) of sex at either age or in either task so all results are collapsed across sex. To avoid effects of repeated exposure, one set of objects was used at PD21 (top), and a distinct set was used at adulthood (bottom). Whether animals experienced the flat/asymmetrical or pointy/symmetrical object was counterbalanced within litters at both ages. Both sets of objects were 3D-printed in polyvinyl (PETG) non-porous material and scaled to either pup (Flat/Asym: 3.9 × 3.6 × 7.9 cm, Pointy/Sym: 3.8 × 8.5 cm) or adult size (Flat/Asym: 14.6 × 8.5 × 5cm, Pointy/Sym: 7.5 × 13 cm). **D–G)** Mean (± SEM) test phase **(D,E)** DRs = [(Time(s)_novel_/Time(s)_novel + familiar_] **(F,G)** time (s) spent rearing in the open (*RIO*), rearing on an object (*ROO*), or Sitting/Climbing On an object (*SCOO*) for (left) pre-weanlings or (right) young adult rats during **(D,F)** NOL or **(E,G)** NOR task. Novelty Preference (DRs): One-sample *t*-test versus chance (0.5) performance indicated by dashed line, #*p* < 0.05, ##*p* < 0.01, ###*p* < 0.001. Flat/asymmetrical versus Pointy/symmetrical (DRs): Independent-samples *t*-test for pairwise comparison between groups, *p < 0.05, **p < 0.01. Flat/asymmetrical versus Pointy/symmetrical, time (s) spent RIO, ROO, SCOO: 2 (object type) × 3 (behavior type) ANOVA with Holm-Šídák’s multiple comparisons *post hoc* test for pairwise comparisons of behavior type main effect, vertical bracket “[” ^#^*p* < 0.05, ^##^*p* < 0.01, ^###^*p* < 0.001, or object × behavior interaction, horizontal bracket “ ⊓ ” *p < 0.05, **p < 0.01, ***p < 0.001. There were no significant main effects of object (all *p’*s > 0.05).

However, additional behavioral analyses revealed a striking developmental difference in exploration behavior between pre-weanling and adult rats during NOL retrieval. When not exploring the objects directly, adult rats (>PD60; Figure 1F, right) spent most of their time rearing in the open arena (RIO) on their hind legs and less time rearing with their paws touching the objects (ROO) or sitting/climbing on the objects (SCOO). This was true for both adult object types. In contrast, pre-weanlings (~PD21, Figure 1F, left) demonstrated a distinct *switch* in their rearing and sitting/climbing behaviors, dependent on which object type was present. When NOL test objects were Flat/Asym, young rats spent significantly more time SCOO than they did RIO or ROO. Interestingly, when the objects were Pointed/Sym, the pattern was reversed; they showed a more adult-like pattern and spent more time RIO than they did SCOO.

We initially hypothesized weaker (relative to flat/asymmetrical) but still significant learning using the pointy/symmetrical object, and that these objects produced no spatial novelty preference at either age was surprising. Single-trial incidental spatial tasks produce weaker memory (i.e., memory deficits beginning at 48 h retentions for some protocols; Reger et al., 2009b) relative to reinforced learning (with memory evident at 30 days or more following a single conditioning trial; Poulos et al., 2016) and so objects that are specifically designed to further reduce spatially relevant information may fail to elicit the significant novel-object preference. This interpretation has alarming implications for researchers who have not examined the characteristics of their objects relative to memory performance. Indeed, unpublished data from our lab using an intermediate object (pointed on top so that it is not sittable/climbable but with an asymmetrical lower shape) elicited intermediate DRs between the pointy and flat objects. This type of parametric morphing between shape designs is an interesting future direction that could generate informative hypotheses regarding the nature of incidental learning with different object shapes.

It could be concluded that, in pre-weanlings, this switch is an obvious effect of the “climb” affordance. However, if this were true, adults should also show the same switch in exploratory behavior. Rather than merely a physical challenge, we posit that this switch reflects a neurobehavioral ontogeny of exploration behavior that facilitates spatial learning in pups with underdeveloped spatial mapping systems. Furthermore, during a non-spatial object identity (NOR) task (Figure 1E), pre-weanlings behaved more like adults; they lacked flat-top-enhanced learning, and object-type exploration differences disappeared (Figure 1G). Developing rats naturally shift their rearing behavior as they age (Shan et al., 2025), however our data show that the behavioral shift is dissociable by psychological process (i.e., spatial versus identity memory). Assessing spatial learning without considering object-type and environmental exploration would miss this critical interplay.

It should be noted that our findings revealed some interesting aspects of *adult* behavior as well. Because testing age was a within-subject factor, we used two distinct object sets in the pre-weanling and adult tests. This within-subject design introduces potential repeat-testing effects that could alter adult behavior relative to naïve adults tested for the first time. An interesting future direction will investigate whether adult behavioral patterns are influenced by developmental learning experiences. Similarly, it is unknown whether the same objects used in pre-weanlings, scaled to adult size, would support similar behavioral outcomes using our design. These are both interesting areas we are currently exploring with additional experimentation.

## Toward richer neurobehavioral phenotyping

Assessing these additional behavioral measures has interesting implications for our understanding of spatial memory development. Self-directed motivation and rearing support information gathering and spatial learning during exploration (Cassity et al., 2024; Concetti et al., 2024; Mun et al., 2015; Shan et al., 2023, 2025) through hippocampus-dependent mechanisms (Layfield et al., 2023). Spatially-tuned cells in the temporal lobe support spatial learning and mature along distinct developmental timelines (Ainge and Langston, 2012; Langston et al., 2010; Moser et al., 2015; Muessig et al., 2015; Raithel and Gottfried, 2021; Tan et al., 2017). Ainge and Langston (2012) discuss the neurophysiological ontogeny of spatial mapping systems, noting significant changes in place-cell and grid-cell numbers and firing characteristics between PD20 and PD29, suggesting ongoing maturation of hippocampal mapping systems during this peri-weaning period. Around PD20-23 is the period in which rats’ place cell distribution and firing patterns change to be more adult-like (Muessig et al., 2015), approximately when rodents first show NOL memory as implemented with the current experimental parameters (Jablonski et al., 2013; Westbrook et al., 2014). While little is known ontogenetically, a low signal-to-noise ratio in spatially tuned cell firing may reduce hippocampal gain modulation that would otherwise facilitate discrimination of fine detail during tests following single-trial learning procedures (Bicanski and Burgess, 2018; Lenkey et al., 2025). Top-down recall of the sample phase environment clashes with bottom-up perceptual input during the test phase (Bicanski and Burgess, 2018) as the object identity and location representations are activated by the hippocampus (Ferguson and Cardin, 2020; Manns and Eichenbaum, 2009). An intriguing possibility, inferred from our data, is that the shift in exploratory behavior between climbable and non-climbable objects in pre-weanlings facilitates fine-tuning of an otherwise noisy spatial mapping system that has only recently begun supporting short-term spatial memory processes. Relative to adults, the young temporal-lobe neurocircuitry may be more sensitive to the additional spatial information afforded to it by rearing/climbing that facilitates stronger mapping (e.g., adult-like regularity and specificity in grid-cell firing patterns). Examining the firing patterns of spatially tuned cells in the extended hippocampal network with and without vertical climbing during NOL *across ontogeny* could reveal whether this is the case and also demonstrate a novel neurobehavioral mechanism by which the developing brain processes spatial information.

Although we have focused our discussion on the development of spatial information processing, it is important to acknowledge that the neural circuitry for visual processing in rodents is also not fully mature at PD21. Early developmental patterns of spontaneous activity that drive circuit development have stopped (Demas et al., 2003) but dorsal and ventral streams and receptive fields have not yet reached full maturity (Binns and Salt, 1997; Smith et al., 2017) and binocularly guided behavior is still developing (Allen et al., 2022). This sensory immaturity could therefore also drive different engagement with objects and their affordances, which could contribute to the observed behavioral effects. This interesting possibility highlights the complexity of studying behavior during the early post-natal period, as multiple neurodevelopmental trajectories are proceeding in parallel.

## Moving beyond summary indices

Animals of different ages may arrive at the same discrimination ratio through fundamentally different behavioral strategies and neural mechanisms. If so, developmental recognition-memory studies may be overlooking some of the most informative aspects of behavior by focusing almost exclusively on novelty preference. Though beyond the scope of the current perspective, Swiercz et al. (2025) discuss whether discrimination ratios/indices actually represent memory or should be reinterpreted to more accurately describe novelty preferences. We agree that an expanded analysis of significant novelty *and* familiarity preferences would contribute to our understanding of incidental learning and whether using “recognition memory” nomenclature in these studies is appropriate when it is not directly tested.

Here, we recommend that, in addition to DR/DI measures of object exploration, developmental studies in particular should include measures of environmental exploration like rearing and climbing during the habituation, sampling, and testing phases. These behaviors show ontogenetic shifts in their patterns that are sensitive to environmental features and object cues (e.g., Shan et al., 2023). Also, as is emerging in fear conditioning studies, measures of activity typically associated with anxiety-like behavior such as thigmotaxis, freezing, darting, and locomotion across habituation, sample, and test phases may reveal developmental changes in how environmental sampling interacts with specific object novelty characteristics (e.g., does thigmotaxis during testing differ between ages in spatial and non-spatial novelty tasks). We do not intend these recommendations to be prescriptive or exhaustive; rather, as a starting point to encourage *novel* thinking around the measures included in these reports.

Future investigations should move beyond asking *whether* an animal remembers, and instead ask *how* memory is expressed, what environmental features shape that expression, and which neural systems support it at different stages of life. Recognition tasks already provide a powerful window into cognitive development; realizing their full potential will require *treating behavior not as a single outcome measure*, but as a rich source of information about the developing brain.

## Data Availability

The original contributions presented in the study are included in the article/supplementary material, further inquiries can be directed to the corresponding author.
